# Growing a greener biomedical community together

**DOI:** 10.1016/j.ebiom.2021.103467

**Published:** 2021-06-24

Since the start of the COVID-19 pandemic, the measures taken in reaction to it presented a mixed environmental impact with both indirect positive and negative effects. Due to global restrictions on travel, there has been a reduction in greenhouse gas emissions. However, the pandemic has also led to an increase in medical waste, including greater use of disposable personal protective equipment and toxic disinfecting chemicals. Early on, prominent environmental activists, such as Nobel laureate economist Joseph Stiglitz in June 2020, suggested to use this unfortunate opportunity to rethink the model of our societies, starting by imagining more sustainable alternatives to our daily activities. Remote working and switching from in-person to virtual conferences are two alternatives that have already been tested in the biomedical community. However, a lot more can be done to align the, currently, negative environmental impact of laboratories and hospitals with their public health mission. With World Environment Day on June 5 and World Oceans Day on June 8 now is the right time to consider some key facts and to share some initiatives with our readers.

According to the international non-governmental organisation *Health care without harm* (Brussels, Belgium, Manila, The Philippines, and Washington, DC, US) the footprint of the health-care sector is equivalent to 4 · 4% of global net emissions, meaning that this sector would be the fifth largest emitter on Earth if it were a country. The sources of emissions range from intensive use of energy and water to hazardous chemicals (eg, for anaesthesia), medical waste, and large amounts of single-use plastic. Similarly, biomedical laboratories with specific equipment (eg, ultra-low temperature freezers and fume hoods) are some of the largest energy and water consuming entities: the non-profit organisation *My Green Lab* (San Diego, CA, US) estimated that research laboratories consume ten times more energy and use four times more water than typical office spaces. Relying heavily on disposable tools, they also generate close to 12 billion pounds of plastic waste per year.

While the negative effects on health due to climate change, greenhouse gases, water scarcity, and contaminated water have been widely documented (see the report of the *Lancet* Countdown on health and climate change), the effects of microplastics (that are shed from plastic waste) on human health are still under investigation. However, their ubiquity and increasing volume is worrisome. The endocrine disrupting chemicals that are carried by some microplastics represent an additional source of health concerns. Microplastics were also recently observed for the first time in the placentas of four out of six human participants using Raman microspectroscopy*.* These studies are an invitation for further investigation and an impetus to take action.

One simple way to act is for researchers to reduce, reuse, and recycle. To encourage colleagues to adopt these rules, some have shared valuable experiences and practical tips. For example, Joana Alves and colleagues (University of Edinburgh, Edinburgh, UK) reported the results of a 4-week test to reduce plastic waste in their own laboratory. Reliance on plastics was reduced by switching from single-use tools to sustainable tools, such as metal versus plastic inoculation loops. To promote reuse over discarding, specific decontamination protocols were designed for commonly used plastic utensils. These strategies allowed them to reduce waste by 44% (43 kg) over 4 weeks. To adjust to each laboratories requirements, managers can tailor their plans of action using the sustainable-lab consumables guide from University College London (London, UK). Beyond tackling the waste problem, laboratories can also act to reduce their consumption of energy and water. Simple solutions, as described in the comprehensive GoGreen guide written by a team of Technische Universität Dresden (Dresden, Germany) participating in the 2017 international Genetically Engineered Machine competition, are already making a difference. An increasing number of institutions are now offering green certification to promote responsible practices in their laboratories (eg, shutting the sash of fume hoods and raising the freezer temperature from –80°C to –70°C). Following suit, *UK Research and Innovation* (an organisation that unites the funding bodies) pledged to integrate green certifications into the grant evaluation process by 2025. While waiting for similar change in other countries, applicants should already include sustainability actions into their proposed research programme.

A growing number of clinicians are also recognising their duty to address this urgent problem and have started analysing the effect of their practices. From these efforts, a new field has been developed: health-care sustainability. In October 2020, the Working Group for Environmental Sustainability in Clinical Care reviewed the state of this field. This group of physicians, public health experts, and environmental health experts recommended a wide range of actions, including documenting the life cycle of medical devices and drugs, revising infection control standards (that drive non-evidence-based use of disposable tools), and forming a global commission tracking the progress made. Audits to quantify waste burden and to identify areas for improvement are becoming more common. For example, a Canadian team of orthopaedists led by Marcia Clark (University of Calgary, Canada) found that, for cases from six orthopaedic subspecialties, an average of 6 · 2kg of waste was collected per case. With 75% of this waste potentially recyclable, the authors recommended implementing appropriate waste segregation and efficient recycling programmes. Such audits might be, by themselves, a tool to reduce cost and waste of procedures as reported by Keon Young Park and colleagues (University of Wisconsin, WI, USA). They observed the positive effects of a monthly surgeon report card detailing the use and cost of disposable and reusable surgical supplies for paediatric laparoscopic procedures: the median cost per case decreased by 43% and the use of disposable tools decreased by 33 to 56% (depending on the type of tools) without a significant change in the clinical outcomes.

The professional life of clinicians and researchers goes beyond the hospital or the laboratory, with conferences representing another major source of greenhouse gas emissions. Olena Zotova and colleagues advocated for greener conferences sharing their experience organising the 2018 annual meeting of the International Federation of Medical Students’ Association (Copenhagen, Denmark). All aspects of a conference, including selecting venues located near public transportation, reducing the use of animal products for catering, prohibiting single use utensils, reducing the amount of merchandise per attendee, and recording the amount of waste generated need to be considered to reach the goal of carbon neutral events. As these measures could substantially change the conference experience, the benefits of such actions should be clearly communicated to the audience.

We can all act to reduce our carbon footprint and we kindly encourage our readers to evaluate the environmental impact of their own activities, in the laboratory, the clinic, and at home. Many small steps can collectively result in large and meaningful change.

*EBioMedicine*

